# Association of Interleukin-23 receptor gene polymorphisms with susceptibility to Crohn’s disease: A meta-analysis

**DOI:** 10.1038/srep18584

**Published:** 2015-12-18

**Authors:** Wang-Dong Xu, Qi-Bing Xie, Yi Zhao, Yi Liu

**Affiliations:** 1 Department of Rheumatology and Immunology, West China Hospital, Sichuan University, 37 Guoxue Road, Chengdu, Sichuan, 610041, PR China

## Abstract

Studies investigating the association between Interleukin-23 receptor (IL-23R) gene polymorphisms and Crohn’s disease (CD) report conflicting results. Thus, a meta-analysis was carried out to assess the association between the IL-23R polymorphisms and CD. A systematic literature search was conducted to identify all relevant studies. Pooled odds ratio (ORs) with 95% confidence interval (CIs) was used to estimate the strength of association. Finally, a total of 60 case-control studies in 56 articles, involving 22,820 CD patients and 27,401 healthy controls, were included in the meta-analysis. Overall, a significant association was found between all CD and the rs7517847 polymorphism (OR = 0.699, 95% CI = 0.659 ~ 0.741, *P* < 0.001). Meta-analysis of the rs11209026, rs1343151, rs10489629 and rs11465804 polymorphisms indicated the same pattern as for rs7517847. Meta-analysis showed an association between the rs10889677A allele and CD (OR = 1.393, 95% CI = 1.328 ~ 1.461, *P* < 0.001). Similarly, meta-analysis of the rs2201840, rs1004819, rs1495965 and rs11209032 polymorphisms revealed the same pattern as that shown by meta-analysis of rs10889677. Stratification by ethnicity revealed that IL-23R gene polymorphisms were associated with CD in the Caucasian group, but not in Asians. In summary, the meta-analysis suggests a significant association between IL-23R polymorphisms and CD, especially in Caucasians.

Crohn’s disease (CD) is a chronic, inflammatory, autoimmune disease characterized by transmural inflammatory lesions that affect the entire gastrointestinal tract^1^. The clear mechanisms of CD pathogenesis remain to be elucidated. Studies in twins and family members indicate that CD is significantly associated with genetic susceptibility^2^,^3^. It has now been accepted that NOD2/CARD15 gene is related to innate immunity in CD^4^,^5^. Furthermore, genes of ATG16L1^6^ and IRGM^7^,^8^ have been recognized to play pivotal roles in autophagy in CD. However, these genetic variants can not fully explain the disease onset of CD. To date, genome-wide association studies have identified approximately 80 CD-susceptibility loci, indicating a series of genes and pathogenic mechanisms, such as microbe recognition, lymphocyte activation, cytokine signaling and intestinal epithelial defense^9^,^10^,^11^. Thus, genetic association studies provide a foundation for the immunopathogenesis for the disease, implicating the role of innate and adaptive immunity in CD occurrence.

Interleukin-23 (IL-23), an important immuno-regulatory cytokine secreted by activated macrophages and dendritic cells, regulates the differentiation of T helper 17 cells from native CD4+ T cells^12^. Binding to IL-23 receptor (IL-23R) complex, IL-23 performed significantly through the Janus kinase (JAK)-signal transducer and activator of transcription (STAT) and NF-κB signaling pathways^13^,^14^. The proportion of IL-23R-expressing T cells in the periphery was 2-fold higher in ankylosing spondylitis (AS) patients than in healthy controls, specifically driven by a 3-fold increase in IL-23R-positive γ/δ T cells in AS patients^15^. γ/δ T cells from AS patients were skewed toward IL-17 production in response to stimulation with IL-23. In IL-23R reporter mice, γ/δ T cells responded to IL-23 during experimental autoimmune encephalomyelitis. IL-23-activated γ/δ T cells rendered αβ effector T cells refractory to the suppressive activity of regulatory T (Treg) cells and also prevented the conversion of conventional T cells into Foxp3(+) Treg cells, suggesting that IL-23/IL23R may play a potential role in autoimmune diseases^16^. IL-23/IL23R signaling plays critical roles in innate and adaptive inflammatory responses in the intestinal mucosa^17^. In mice models, IL-23/IL-23R, a key component of IL-23/Th17 pathway, is essential for intestinal inflammation of T cell-dependent colitis^18^,^19^. Furthermore, patients with CD manifested increased IL-23 secretion compared with controls^20^. A genome-wide association study indicated that IL-23R gene is a potential candidate for CD susceptibility^21^. IL-23R gene is mapped to the chromosome 1 (1p31.3)^13^. Recently, polymorphisms in this locus have been identified and a large number of studies indicated the association between these polymorphisms and CD risk^21^,^22^,^23^,^24^,^25^,^26^,^27^,^28^,^29^,^30^,^31^,^32^,^33^,^34^,^35^,^36^,^37^,^38^,^39^,^40^,^41^,^42^,^43^,^44^,^45^,^46^,^47^,^48^,^49^,^50^,^51^,^52^,^53^,^54^,^55^,^56^,^57^,^58^,^59^,^60^,^61^,^62^,^63^,^64^,^65^,^66^,^67^,^68^,^69^,^70^,^71^,^72^,^73^,^74^,^75^,^76^. However, previous studies in different populations reported conflicting results. The disaccord may be attributed to small sample size, various racial and ethnic backgrounds, uncorrected multiple hypothesis testing and publication bias.

Meta-analysis is a statistical method for augmenting the effective sample size through combining the results of several studies to produce a single estimate of the major effect with enhanced precision. It is considered to be a powerful tool for pooling inconsistent results from different studies^77^. To date, two meta-analysis^52^,^78^ of the association between the IL-23R gene polymorphisms and CD risk have been reported, but these results were still inconclusive. New studies about the role of these SNPs in the IL-23R gene in CD have been published in recent years^53^,^54^,^55^,^56^,^57^,^58^,^59^,^60^,^61^,^62^,^63^,^64^,^65^,^66^,^67^,^68^,^69^,^70^, and these studies might provide new evidence. Thus, it seems necessary to perform an updated and comprehensive meta-analysis including the latest data to investigate the association of the IL-23R gene polymorphisms and the risk of CD.

## Materials and Methods

### Publication research

A literature search was conducted for studies that examined association between the IL-23R gene polymorphisms and CD risk in October 13, 2015. We used PubMed database, Elsevier Science Direct, China National Knowledge Infrastructure database (CNKI) and Chinese Biomedical database (CBM) to identify articles with the following terms: “Interleukin-23 receptor, IL-23R, Crohn’s disease, Crohn disease, inflammatory bowel disease, CD and IBD”. References in the studies were also reviewed to find additional studies. The languages were limited to English and Chinese.

### Inclusion and exclusion criteria

A study was included in the analysis if a) it was a case-control or cohort study; b) it included original data (independence among studies); and c) it provided sufficient data to calculate the odds ratio (OR). We excluded the followings: a) studies contained overlapping data; b) studies in which family members had been studied because of the analysis based on linkage considerations.

### Data extraction

Data were collected by two independent investigators (Qi-Bing Xie and Yi Zhao). The characteristics of the selected articles were in Table 1, including first author, year of publication, study population, ethnicity, numbers of case and control, genotyping method, the concrete polymorphisms investigated in these studies. The study populations comprised Italian, German, Dutch, Hungarian, Korean, Chinese, Malaysian, African American, Australian, Canadian, Algerian and so on. The Asian subgroup included Korean, Chinese, and Malaysian populations. African American population was classified in the African subgroup and others in the Caucasian subgroup.

### Statistical analysis

Allele frequencies at the IL-23R gene polymorphisms from the respective study were determined by the allele counting method. The contrast of the allelic effect of minor allele versus common allele of the IL-23R gene polymorphisms was tested. The strength of association between the IL-23R gene polymorphisms and CD susceptibility was assessed by odds ratios (OR) and 95% confidence intervals (95% CI).

The heterogeneity of between-studies was evaluated by the Chi-square test based Q-statistic^79^. In addition, the I^2^-statistic also measures the degree of inconsistency in the studies by computing what percentage of the total variation across studies was due to heterogeneity rather than by chance. A high value of I^2^ indicated a higher probability of the existence of heterogeneity (I^2^ = 0% to 25%, no heterogeneity; I^2^ = 25% to 50%, moderate heterogeneity; I^2^ = 50% to 75%, large heterogeneity; and I^2^ = 75% to 100%, extreme heterogeneity). If a *P*-value of the heterogeneity Q-statistic was more than 0.10, the pooled OR was calculated by the fixed effects model. Otherwise, a random effects model was adopted. Potential publication bias was analyzed by Egger’s linear regression test and the Funnel plot. If *P*-value was less than 0.05, statistically significant publication bias might exist^80^.

All the statistical analysis of meta-analysis was performed by STATA statistical software (version 11.0 STATA Corp LP, College Station, TX, USA).

## Results

### Study characteristics

**Figure 1:**
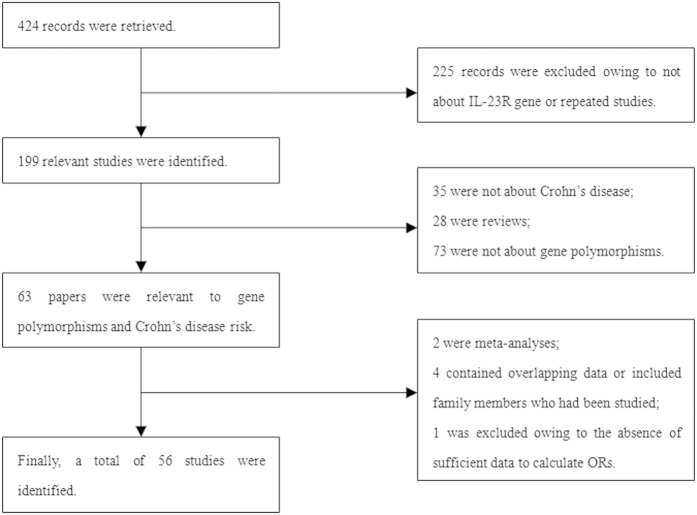
Process of selecting studies.

The process for selecting the studies was shown in Fig. 1. 424 potentially relevant records were first reviewed, and 56 articles met the inclusion criteria were finally included in themeta-analysis^21^,^22^,^23^,^24^,^25^,^26^,^27^,^28^,^29^,^30^,^31^,^32^,^33^,^34^,^35^,^36^,^37^,^38^,^39^,^40^,^41^,^42^,^43^,^44^,^45^,^46^,^47^,^48^,^49^,^50^,^51^,^52^,^53^,^54^,^55^,^56^,^57^,^58^,^59^,^60^,^61^,^62^,^63^,^64^,^65^,^66^,^67^,^68^,^69^,^70^,^71^,^72^,^73^,^74^,^75^,^76^. Among the 56 articles, 4 were published in Chinese and the others in English. Of the 56 articles, one article included three cohorts^24^, other two^21^,^68^ included two cohorts, thus, each cohort was considered as a single study. Finally, a total of 60 case-control studies in 56 articles involving 22,820 CD patients and 27,401 healthy controls were identified. There were 49 studies on rs11209026, 25 studies on rs7517847, 19 studies on rs2201841 and rs1004819 separately, 18 studies on rs10889677, 15 studies on rs1343151 and rs1495965 separately, 13 studies on rs11209032, 12 studies on rs10489629 and 8 on rs11465804. Forty-nine studies involved Caucasian populations^21^,^22^,^23^,^24^,^25^,^26^,^27^,^28^,^29^,^30^,^31^,^32^,^33^,^34^,^35^,^36^,^37^,^38^,^39^,^40^,^41^,^42^,^43^,^44^,^45^,^46^,^47^,^48^,^49^,^50^,^51^,^52^,^53^,^54^,^55^,^56^,^57^,^58^,^59^,^60^,^61^,^62^,^63^,^64^,^66^,^67^,^68^,^69^, nine studies involved Asian populations^34^,^51^,^61^,^70^,^71^,^72^,^73^,^74^,^75^, and two study involved African populations^65^,^76^. The main characteristics of each study included in this meta-analysis were summarized in Table 1.

### Evaluation of heterogeneity and publication bias

Between-study heterogeneity was found in some meta-analysis of the IL-23R gene polymorphisms in CD (Table 2). Therefore, these meta-analyses were performed in a random effects model, and the other meta-analyses were done in a fixed effects model.

The funnel plot asymmetry and Egger’s test were performed to assess potential publication bias. If there was asymmetry, the index to determine Egger’s test will show *P* < 0.05. It was shown that there was no significant publication bias between all of the comparisons (all *P*-value > 0.05).

### Meta-analysis of IL-23R gene polymorphisms in Crohn’s disease

A summary of the meta-analysis of the association between the IL-23R gene polymorphisms and CD was listed in Table 2.

### IL-23R rs7517847, rs1343151, rs10489629 polymorphisms and CD

**Figure 2:**
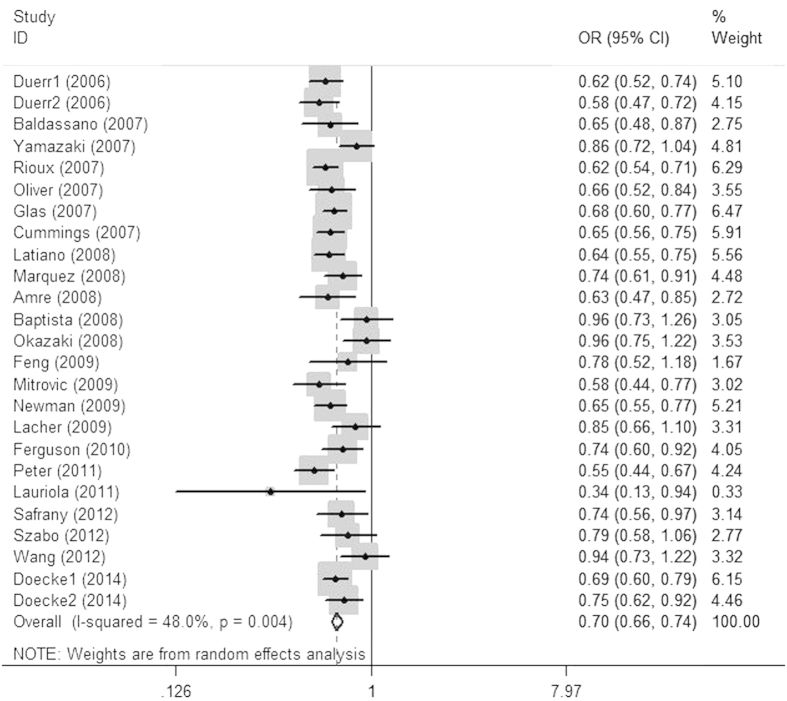
Odds ratios and 95% confidence intervals for individual studies and pooled data for the association between the G versus T allele of the IL-23R rs7517847 polymorphism and Crohn’s disease.

Twenty-five studies determined the relationship between the rs7517848G/T polymorphism and CD risk^21^,^22^,^25^,^27^,^30^,^31^,^34^,^35^,^36^,^41^,^42^,^43^,^47^,^48^,^49^,^54^,^59^,^60^,^63^,^64^,^65^,^68^,^73^. The total sample size for patients with CD and healthy controls was 9,297 and 12,643, respectively. Meta-analysis revealed an association between the rs7517847G allele and CD risk in the overall population (OR = 0.699, 95% CI = 0.659 ~ 0.741, *P* < 0.001; Fig. 2). Stratification by ethnicity indicated that the rs7517847G allele was significantly associated with CD risk in the Caucasian population (OR = 0.669, 95% CI = 0.641 ~ 0.698, *P* < 0.001), but not in the other subgroups. Meta-analysis of the rs1343151 and rs10489629 polymorphisms showed the same pattern as for rs7517847. Minor alleles of all polymorphisms might be protective alleles for CD susceptibility in Caucasians, but not in the other populations (Table 2).

### IL-23R rs10889677, rs1004819, rs1495965, rs11209032 polymorphisms and CD

**Figure 3:**
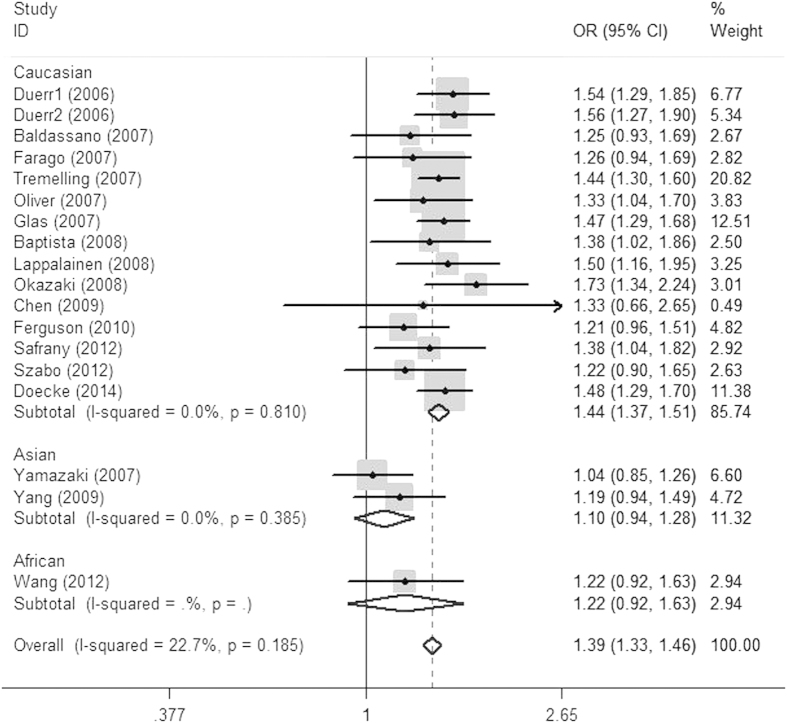
Odds ratios and 95% confidence intervals for individual studies and pooled data for the association between the A versus C allele of the IL-23R rs10889677 polymorphism and Crohn’s disease.

Eighteen studies containing 7,518 cases and 8,671 healthy controls examined the association of rs10889677A/C and CD^21^,^22^,^26^,^27^,^30^,^33^,^34^,^36^,^40^,^43^,^51^,^54^,^63^,^64^,^65^,^68^,^72^. Results indicated a significant association between the rs100889677A/C polymorphism and CD (OR = 1.393, 95% CI = 1.328 ~ 1.461, *P* < 0.001; Fig. 3). Stratifying by ethnicity, we found a significant association in the Caucasian population (OR = 1.438, 95% CI = 1.366 ~ 1.513, *P* < 0.001; Fig. 2). Results by meta-analysis of the rs1004819, rs1495965 and rs11209032 polymorphisms revealed the same pattern as for rs10889677. Minor alleles of the SNPs above might be risk alleles for CD in Caucasian subgroup. Detail results were presented in Table 2.

### IL-23R rs2201841, rs11209026, rs11465804 polymorphisms and CD

**Figure 4:**
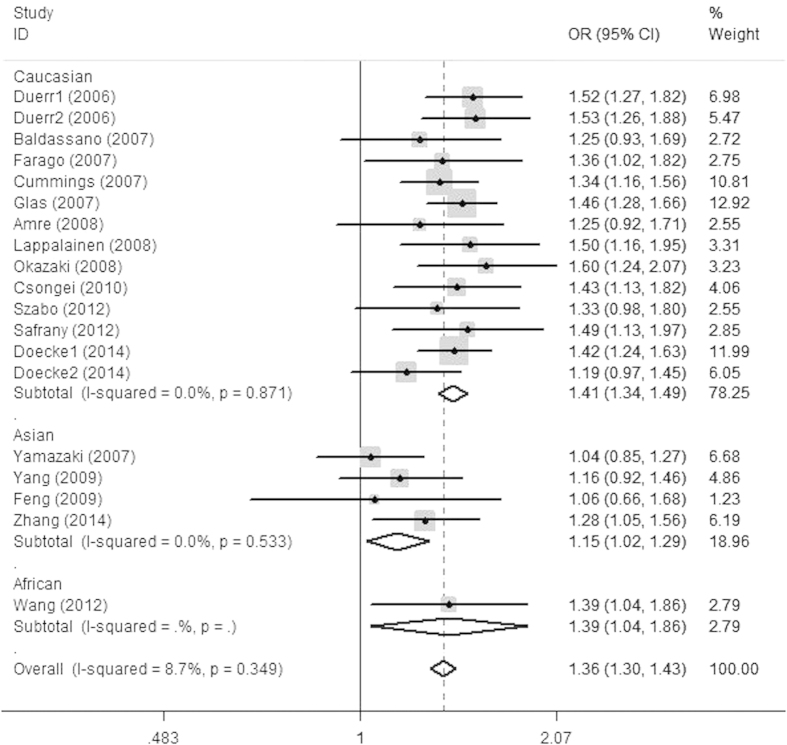
Odds ratios and 95% confidence intervals for individual studies and pooled data for the association between the C versus T allele of the IL-23R rs2201841 polymorphism and Crohn’s disease.

Eighteen case-control studies including 6,846 cases and 9,056 healthy controls identified a significant association between the rs2201841C/T polymorphism and susceptibility to CD^21^,^22^,^25^,^26^,^27^,^34^,^35^,^40^,^43^,^51^,^53^,^63^,^64^,^65^,^68^,^73^. The pooled OR (95% CI, P value) in the C versus T allele was 1.368 (1.301 ~ 1.438, *P* < 0.001). Ethnicity-specific analysis showed that the rs2201841C allele was significantly associated with CD in the Caucasian and African subjects (OR = 1.413, 95% CI = 1.338 ~ 1.491, *P* < 0.001; OR = 1.392, 95% CI = 1.042 ~ 1.858, *P* = 0.025, respectively). The forest plot was shown in Fig. 4. Meta-analysis of the rs11209026 polymorphism showed the same pattern as for rs2201841. In addition, a significant association was found between the rs11465804G allele and the risk of CD in Caucasians (OR = 0.435, 95% CI = 0.376 ~ 0.503, *P* < 0.001) (Table 2). However, Asians and Africans-based subgroup analysis were not performed.

## Discussion

A genome-wide association study by Duerr *et al.*^21^ found strong correlation between IL-23R polymorphisms and inflammatory bowel disease (IBD). Since then, genetic variants of IL-23R have been investigated in numerous autoimmune diseases. It has been shown that the IL-23R gene plays an important role in the pathogenesis of some autoimmune diseases, such as IBD, AS, psoriasis, rheumatoid arthritis, and multiple sclerosis^81^. Oliver *et al.*^30^ and Baptista *et al.*^36^ found rs11209026, rs10889677 and rs1004819 polymorphisms were associated with CD disease susceptibility in Spanish. Similarly, a study on Hungarian with CD revealed that the rs1004819T carrier (TT + TC) had a 53% increasing risk when compared with the CC homozygote^53^. Furthermore, a study in Korean population indicated that the rs1495965G allele frequency was higher in CD cases compared to controls (OR = 1.31, 95% CI = 1.07 ~ 1.60)^51^. However, Okazaki *et al.*^43^ and Chua *et al.*^61^ found no significant differences in allele or genotype frequency of rs11209026 and rs1004819 polymorphisms between CD patients and controls in a Malaysian population.

Therefore, to better comprehend the association between polymorphisms in the IL-23R gene and CD susceptibility, a pooled analysis with a larger sample size, subgroup analysis performed and heterogeneity explored is needed. Overall, results of this meta-analysis suggested that IL-23R gene polymorphisms were associated with CD susceptibility (all *P* value < 0.001). Ethnicity-specific analysis showed that the polymorphisms in the IL-23R gene might confer susceptibility to CD in Caucasians, but not in Asians.

The diverse roles of the same gene polymorphism in ethnicity-specific analysis by ethnicity could be ascribed to the following major aspects. First, Yamazaki *et al.*^34^ showed that IL-23R gene was not a candidate gene to CD in the Japanese population. Furthermore, Lee *et al.*^82^ found that rs1004819, rs10489629, rs1343151, rs1495965, rs11209032 and rs2201841 polymorphisms revealed a different association with ankylosing spondylitis between Caucasians and Asians. Compared to the populations of Caucasian, no significant association was detected for IL-23R polymorphisms in Chinese psoriasis patients^83^. Similarly, IL-23R (rs1004819, rs7517847, rs10489629, rs2201841, rs1343151, rs11209032, and rs1495965) polymorphisms were not associated with systemic lupus erythematosus (SLE) in the Korean population^84^, while rs10889677 and rs7517847 polymorphisms were not associated with Chinese SLE patients^85^. In addition, CD is the most prevalent in North America and Europe, and the least prevalent among African Americans and Asians^86^. These findings suggested that IL-23R gene might have a susceptible nature in the Caucasians, but not in the Asians. Therefore, the inconsistent results of subgroup analysis might be attributed to ethnic differences. Second, for other variants in the IL-23R gene, Zhao *et al.*^75^ found that IL-23R rs11805303 and rs17375018 polymorphisms were not associated with CD disease susceptibility in Chinese. Similarly, a study in another Chinese population showed no significant differences in allele or genotype frequency of the rs11805303 polymorphism between CD patients and controls^71^. However, Chen *et al.*^72^ found that the rs11465788T allele frequency was lower in Chinese CD cases compared to controls (OR = 0.30, 95%CI = 0.15–0.60). Since few studies in Asian population focus on these polymorphisms, these results might not be reliable. Therefore, large sample size GWAS should be considered in future to confirm the role of these variants in the IL-23R gene in Asians. Third, most autoimmune diseases including CD are multifactor diseases, and these diseases were caused by an interaction of genetic and environmental factors^87^. Therefore, different populations lived in different environments, gene-environment interactions may partly affect the autoimmune diseases susceptibility. It has been widely accepted that genetic and environmental factors play an important role in disease initiation such as SLE, and the progression of the disease. A previous study has confirmed that the IFIH1 rs1990760 polymorphism was associated with SLE in the European population, not in the Asian population^88^, suggesting that interactions between different environments and genes might be different.

Although the IL-23R gene is associated with CD risk, the function of IL-23R gene variants remains unclear. Several possible mechanisms can be suggested by which polymorphisms can regulate the function of the IL-23R gene. First, the standard form of IL-23R is encoded by at least 12 exons. Zhang *et al.*^89^ demonstrated that at least six spliced isoforms of IL-23R (IL-23R1 to 6) can be generated through alternative splicing. Translation prediction revealed that spliced variants led to either premature termination to give rise to a diverse form of receptor ectodomain, or a frameshift to generate various lengths of the IL-23R endodomain^89^. Furthermore, the intronic polymorphisms, such as rs11805303, rs1004819, might exert their influence by regulating differential splicing. Second, the rs11209026 polymorphism, also named as Arg381Gln, is located between the transmembrane domain and the putative JAK2 binding site in the cytoplasmic portion of IL23R protein and highly conserved between species^90^. Replacing conserved Arg381 for Gln381 at this position probably modulates IL-17 and IL-22 expression in response to IL-23 stimulation, which may have a functional influence on IL-23R signaling pathway^91^,^92^. Third, the rs10889677 polymorphism, located in the 3′-UTR, might promote overexpression of the receptor through increasing mRNA stability and driving T cells to differentiate towards Th17, and thus, leading to inflammation by increasing release of other cytokines^81^. Forth, the rs10889677 variant enhanced both mRNA and protein expression of IL-23R can lead to a loss of binding capacity to the microRNAs (miRNAs) Let-7e and Let-7f. MiRNA-mediated dysregulation of IL-23R signaling correlated with a single nucleotide polymorphism in the IL-23R gene was strongly associated with CD susceptibility, suggesting that this mutation can lead to altered IL-23R signaling^93^.

With respect to the IL-23R haplotypes and CD, Taylor *et al.*^94^ showed that haplotype TA (rs1004819 (T) + rs790631 (A)) was 55.4% in the controls, and 64% in CD patients (P = 0.019), indicating a haplotype inferring increased CD risk. Similarly, haplotype CACCGC (rs7530511 (C) + rs7528924 (A) + rs2201841 (C) + rs10489628 (C) + rs11209026 (G) + rs1343151 (C)) was 55.8% in the controls, and 64.4% in CD patients (P = 0.013), indicating a “risk” haplotype. On the contrary, haplotype GA (rs1004819 (G) + rs790631 (A)) was 64.5% in the control group, and 54.4% in CD patients (P = 0.006), suggesting a haplotype inferring decreased CD risk. Similarly, haplotype CATTGT (rs7530511 (C) + rs7528924 (A) + rs2201841 (T) + rs10489628 (T) + rs11209026 (G)+ rs1343151 (T)) was 47.0% in the controls, and 36.6% in CD patients (P = 0.001), suggesting a “protective” haplotype. In addition, Szabo *et al.*^64^ found that haplotype GTTTAC (rs1004819 (G) + rs7517847 (T) + rs7530511 (T) + rs2201841 (T) + rs1343151 (A) + rs10889677 (C)) showed a strong protective effect in CD, and it reduced disease risk (OR = 0.37, 95% CI = 0.17 ~ 0.78, *P* = 0.009). Furthermore, Doecke *et al.*^68^ reported that haplotype GG (rs1004819 (G) + rs7517847 (G)) was negatively related to CD risk (OR = 0.71, 95% CI = 0.62 ~ 0.81, *P* < 0.001). However, haplotype AT (rs1004819 (G) + rs7517847 (G)) was positively related to CD risk (OR = 1.45, 95% CI = 1.26 ~ 1.67, *P* < 0.001).

Compared with the previous meta-analysis^52^,^78^, the current study involved a total of 60 case-control studies in 56 articles, which is much larger than the data of the previous meta-analysis. Moreover, we performed subgroup analysis by ethnicity to discuss the ethnic effect on the risk of CD. Thus, our meta-analysis might enhance the statistical power and draw a more reliable conclusion.

Some limitations of the present study should be considered. First, we could not analyze the potential gene-environment interactions and gene susceptibility haplotypes owing to lack of data, such as the data of environmental risk factors and genotypes. Second, our literature search was only dependent on English and Chinese, language bias might be considered. Third, potential publication bias was not found by statistical method, but it might exist because of only published articles included. Fourth, only one published studies in the African origin was included in the meta-analysis, the stratified analysis for Africans might not be reliable. Thus, the results were applicable only to the Asian and Caucasian groups. Finally, different genotyping methods and disease status might affect the data interpretation of the included studies.

In summary, the current study provides a comprehensive examination of the available evidence for the association between polymorphisms in the IL-23R gene and CD. This updated meta-analysis suggests that IL-23R gene polymorphisms are associated with CD susceptibility. However, larger sample size studies taking environmental risk factors into account and including more ethnic groups should be considered in future to confirm the results from our meta-analysis.

## Additional Information

**How to cite this article**: Xu, W.-D. *et al.* Association of Interleukin-23 receptor gene polymorphisms with susceptibility to Crohn,s disease: A meta-analysis. *Sci. Rep.* **5**, 18584; doi: 10.1038/srep18584 (2015).

## Figures and Tables

**Table 1:** Characteristics of the individual studies included in the meta-analysis.

First Author	Year	Population (Ethnicity)	Numbers	Genotyping Methods	IL-23R polymorphisms studied
			CD/Control		
Duerr^21^	2006	Non-Jewish (C)	547/548	Sequencing	rs1004819, rs7517847, rs10489629, rs2201841, rs11465804,
		Jewish (C)	401/433	Sequencing	rs11209026, rs1343151, rs10889677, rs11209032, rs1495965
Baldassano^22^	2007	American (C)	142/281	Sequencing	rs1004819, rs7517847, rs10489629, rs2201841, rs11465804, rs11209026, rs1343151, rs10889677, rs11209032, rs1495965
Borgiani^23^	2007	Italian (C)	187/194	TaqMan	rs11209026
Bunning^24^	2007	German (C)	318/428	PCR	rs11209026
		Hungarian (C)	148/200	PCR	rs11209026
		Dutch (C)	157/217	PCR	rs11209026
Cummings^25^	2007	British (C)	604/1149	Sequencing	rs1004819, rs7517847, rs10489629, rs2201841, rs11209026, rs1343151, rs11209032, rs1495965
Farago^26^	2007	Hungarian (C)	190/220	PCR-RFLP	rs10889677, rs2201841
Glas^27^	2007	German (C)	833/1381	PCR	rs1004819, rs7517847, rs10489629, rs2201841, rs11465804, rs11209026, rs1343151, rs10889677, rs11209032, rs1495965
Leshinsky-Silver^28^	2007	Israeli (C)	282/157	PCR	rs11209026
Limbergen^29^	2007	Scotchman (C)	233/342	TaqMan	rs11209026
Oliver^30^	2007	Spanish (C)	238/342	TaqMan	rs1004819, rs7517847, rs10489629, rs11209026, rs1343151, rs10889677, rs11209032, rs1495965
Rioux^31^	2007	American (C)	988/1007	Sequencing	rs7517847
Roberts^32^	2007	New Zealander (C)	496/591	TaqMan	rs11209026
Tremelling^33^	2007	British (C)	1902/1345	Sequencing	rs1004819, rs10489629, rs11465804, rs11209026, rs1343151, rs10889677, rs11209032, rs1495965
Yamazaki^34^	2007	Japanese (A)	484/439	TaqMan PCR-RFLP	rs1004819, rs7517847, rs10489629, rs2201841, rs11465804, rs11209026, rs1343151, rs10889677, rs11209032, rs1495965
Amre^35^	2008	Canadian (C)	259/139	FP-SBE	rs1004819, rs11209026, rs7517847, rs10489629, rs2201841, rs11465804, rs1343151, rs10889677, rs11209032, rs1495965
Baptista^36^	2008	Brazilian (C)	187/255	TaqMan	rs1004819, rs7517847, rs11209026, rs1495965, rs10889677
Civitavecchia^37^	2008	Italian (C)	199/100	Sequencing	rs11209026
Gaj^38^	2008	Polish (C)	60/139	TaqMan	rs11209026
Lakatos^39^	2008	Hungarian (C)	266/149	PCR	rs11209026
Lappalainen^40^	2008	Finnish (C)	238/292	PCR	rs1004819, rs10489629, rs2201841, rs11465804, rs11209026, rs1343151, rs10889677, rs11209032
Latiano^41^	2008	Italian (C)	723/716	TaqMan	rs11209026, rs7517847
Lu^74^	2008	Chinese (A)	74/100	PCR	rs11209026
Marquez^42^	2008	Spanish (C)	344/547	TaqMan	rs11209026, rs7517847
Okazaki^43^	2008	Canadian (C)	213/310	TaqMan	rs7517847, rs2201841, rs11209026, rs10889677, rs1495965
Venegas^44^	2008	Chilean (C)	38/58	PCR-RFLP	rs11209026
Weersma^45^	2008	Dutch (C)	1684/1350	TaqMan	rs11209026
Chen^71^	2008	Chinese (A)	41/50	PCR	rs11209026
Dusatkova^46^	2009	Czech (C)	333/499	TaqMan	rs11209026
Chen^72^	2009	Chinese (A)	50/50	Sequencing	rs10889677
Feng^73^	2009	Chinese (A)	96/96	Sequencing	rs1004819, rs7517847, rs2201841
Lacher^47^	2009	German (C)	221/253	TaqMan	rs11209026, rs7517847
Mitrovi^48^	2009	Slovenian (C)	159/345	PCR-RFLP	rs7517847
Newman^49^	2009	Canadian (C)	443/1005	Sequencing	rs11209026, rs7517847
Weersma^50^	2009	Dutch-Belgian (C)	1656/1086	Sequencing	rs11209026
Yang^51^	2009	Korean (A)	380/380	Sequencing	rs1004819, rs2201841, rs11209026, rs10889677, rs1495965
Cotterill^52^	2009	British (C)	295/877	Sequencing	rs11209026
Csöngei^53^	2010	Hungarian (C)	315/314	PCR-RFLP	rs1004819, rs2201841
Ferguson^54^	2010	New Zealander (C)	339/407	TaqMan	rs10889677, rs11209026, rs1343151, rs7517847
Gazouli^55^	2010	Greek (C)	474/539	PCR	rs11209026
Mahurkar^56^	2010	Indian (C)	241/442	Sequencing	rs11209026
Sventoraityte^57^	2010	Lithuanian (C)	57/186	TaqMan	rs11209026
Wagner^58^	2010	Australian (C)	72/98	PCR	rs11209026
Lauriola^59^	2010	Italian (C)	19/20	PCR	rs11209026, rs7517847
Peter^60^	2011	Ashkenazi (C)	369/503	TaqMan	rs11209026, rs7517847
Zhao^75^	2011	Chinese (A)	43/134	SNaPshot	rs1343151, rs11209032
Chua^61^	2012	Malaysian (A)	80/100	PCR-RFLP	rs1004819
Jung^62^	2012	Franch (C)	798/960	Sequencing	rs11209026
Safrany^63^	2012	Hungarian (C)	199/253	PCR-RFLP	rs7517847, rs2201841, rs10889677, rs11209032
Szabo^64^	2012	Hungarian (C)	190/182	PCR-RFLP	rs1004819, rs7517847, rs2201841, rs1343151, rs10889677
Wang^65^	2012	African American	354/354	TaqMan	rs7517847, rs2201841, rs11209026, rs1496965, rs10889677
Mihaljevi^66^	2013	Croatian (C)	50/99	PCR	rs11209026
Ballester^67^	2013	Puerto Rican (C)	406/504	Sequencing	rs11209026
Doecke^68^	2014	Australian (C)	675/1255	Sequencing	rs1004819, rs7517847, rs10489629, rs2201841, rs11465804, rs11209026, rs1343151, rs10889677, rs11209032, rs1495965
		New Zealander (C)	318/533	Sequencing	rs1004819, rs7517847, rs10489629, rs2201841, rs11209026, rs1343151
Meddour^69^	2014	Algerian (C)	204/201	TaqMan	rs11209026
Zhang^70^	2014	Chinese (A)	420/450	TaqMan	rs11209026, rs1004819, rs1495965
Huang^76^	2015	African American	1088/1797	Immunochip	rs11209026

CD: Crohn’s disease, C: Caucasian, A: Asian.

**Table 2:** Meta-analysis of IL-23R polymorphisms in CD.

Polymorphisms	Population	No. of studies	Test of association				Test of heterogeneity			Egger’s test (*p*)
			OR (95% CI)	Z	*p*	Model	χ^2^	*p*	I^2^(%)	
rs11209026 A versus G	Overall	49	0.407(0.378 ~ 0.439)	23.32	<0.001	R	75.51	0.007	36.4	0.420
	Caucasian	44	0.406(0.365 ~ 0.452)	16.50	<0.001	R	67.45	0.010	36.2	0.606
	Asian	3	0.659(0.409 ~ 1.063)	1.71	0.087	F	0.47	0.791	0	0.059
	African	2	0.584(0.375 ~ 0.911)	2.37	0.018	F	1.00	0.318	0	NA
rs7517847 G versus T	Overall	25	0.699(0.659 ~ 0.741)	11.93	<0.001	R	46.18	0.004	48.0	0.303
	Caucasian	22	0.669(0.641 ~ 0.698)	18.68	<0.001	F	26.22	0.198	19.9	0.545
	Asian	2	0.896(0.773 ~ 1.039)	1.46	0.145	F	0.42	0.517	0	NA
	African	1	0.940(0.726 ~ 1.216)	0.47	0.637	NA	NA	NA	NA	NA
rs10889677 A versus C	Overall	18	1.393(1.328 ~ 1.461)	13.65	<0.001	F	21.98	0.185	22.7	0.165
	Caucasian	15	1.438(1.366 ~ 1.513)	13.96	<0.001	F	9.32	0.810	0	0.196
	Asian	2	1.098(0.945 ~ 1.276)	1.22	0.222	F	0.75	0.385	0	NA
	African	1	1.220(0.916 ~ 1.626)	1.36	0.174	NA	NA	NA	NA	NA
rs1004819 T versus C	Overall	19	1.352(1.264 ~ 1.446)	8.82	<0.001	R	35.77	0.008	49.7	0.158
	Caucasian	14	1.441(1.373 ~ 1.514)	14.68	<0.001	F	8.73	0.793	0	0.943
	Asian	5	1.102(0.994 ~ 1.222)	1.84	0.066	F	5.86	0.210	31.8	0.242
rs2201840 C versus T	Overall	18	1.368(1.301 ~ 1.438)	12.33	<0.001	F	19.28	0.313	11.8	0.342
	Caucasian	14	1.413(1.338 ~ 1.491)	12.53	<0.001	F	7.56	0.871	0	0.617
	Asian	3	1.088(0.943 ~ 1.255)	1.15	0.250	F	0.53	0.769	0	0.996
	African	1	1.392(1.042 ~ 1.858)	2.24	0.025	NA	NA	NA	NA	NA
rs1343151 T versus C	Overall	15	0.725(0.690 ~ 0.763)	12.49	<0.001	F	19.03	0.164	26.4	0.576
	Caucasian	13	0.717(0.681 ~ 0.755)	12.73	<0.001	F	11.97	0.448	0	0.842
	Asian	2	1.069(0.790 ~ 1.444)	0.43	0.667	F	0.47	0.494	0	NA
rs1495965 G versus A	Overall	15	1.197(1.103 ~ 1.299)	4.29	<0.001	R	43.42	<0.001	67.8	0.129
	Caucasian	12	1.218(1.111 ~ 1.335)	4.20	<0.001	R	34.76	<0.001	68.4	0.180
	Asian	3	1.119(0.945 ~ 1.324)	1.31	0.191	R	4.75	0.093	57.9	0.354
rs11209032 A versus G	Overall	13	1.343(1.279 ~ 1.411)	11.73	<0.001	F	11.51	0.485	0	0.182
	Caucasian	11	1.359(1.291 ~ 1.431)	11.68	<0.001	F	9.16	0.517	0	0.308
	Asian	2	1.181(0.995 ~ 1.403)	1.90	0.057	F	0.02	0.885	0	NA
rs10489629 G versus A	Overall	12	0.791(0.706 ~ 0.887)	4.02	<0.001	R	56.15	<0.001	80.4	0.220
	Caucasian	11	0.775(0.690 ~ 0.869)	4.33	<0.001	R	48.83	<0.001	79.5	0.310
	Asian	1	0.998(0.815 ~ 1.222)	0.02	0.986	NA	NA	NA	NA	NA
rs11465804 G versus T	Overall	8	0.435(0.376 ~ 0.503)	11.18	<0.001	F	6.29	0.506	0	0.082
	Caucasian	8	0.435(0.376 ~ 0.503)	11.18	<0.001	F	6.29	0.506	0	0.082

CD: Crohn’s disease, R: random effects model, F: fixed effects model, NA: not available.
